# Risk factors associated with low bone mineral density in children with idiopathic scoliosis: a scoping review

**DOI:** 10.1186/s12891-023-06157-8

**Published:** 2023-01-20

**Authors:** Yuqi Yang, Zhengquan Chen, Zefan Huang, Jing Tao, Xin Li, Xuan Zhou, Qing Du

**Affiliations:** 1 College of Global Public Health, New York University, New York, NY 10003 USA; 2 Department of Rehabilitation, Xinhua Hospital, School of Medicine, Shanghai Jiaotong University, 1665 Kongjiang Road, Shanghai, 200092 China; 3 Chongming Hospital, Shanghai University of Medicine & Health Sciences, Shanghai, 202150 China

**Keywords:** Idiopathic Scoliosis, Bone Mineral Density, Osteoporosis, Etiology

## Abstract

**Background:**

Children with idiopathic scoliosis (IS) have a high risk of osteoporosis and IS with low bone mineral density (BMD) are susceptible to curve progression. This review aims to explore the risk factors of low BMD in children with IS.

**Methods:**

Studies were retrieved from 5 databases that were published up to January 2022. Search terms are keywords in titles or abstracts, including subject headings related to “Scoliosis”, “Bone Mineral Density”, and “Risk Factors”. Observational studies on risk factors of low BMD in children with IS were enrolled in this review. The number of studies, sample size, outcome measures, research type, endocrine, and lifestyle-related factors, gene/signal pathway, and other contents were extracted for qualitative analysis.

**Results:**

A total of 56 studies were included in this scoping review. Thirty studies involved genetic factors that may affect BMD, including the Vitamin-D receptor gene, RANK/RANKL signal pathway, the function of mesenchymal stem cells, Runx2, Interleukin-6 (IL-6), and miR-145/β-catenin pathway. Eight studies mentioned the influence of endocrine factors on BMD, and the results showed that serum levels of IL-6, leptin and its metabolites, and ghrelin in children with IS were different from the age-matched controls. In addition, there were 18 articles on lifestyle-related factors related to low BMD in children with IS, consisting of physical activity, calcium intake, Vitamin D level, and body composition.

**Conclusions:**

Genetic, endocrine, and lifestyle-related factors might relate to low BMD and even osteoporosis in IS. To prevent osteoporosis, the effectiveness of regular screening for low BMD risk factors in children with IS needs to be investigated. Additionally, clear risk factors suggest strategies for bone intervention. Future studies should consider the effectiveness of calcium and vitamin D supplements and physical activity in BMD improvement.

**Supplementary Information:**

The online version contains supplementary material available at 10.1186/s12891-023-06157-8.

## Background

Idiopathic scoliosis (IS) is the most common type of scoliosis, defined as a three-dimensional structural deformity of the spine, and can be diagnosed when the Cobb angle is ≥ 10 [1]. The prevalence of IS varies widely worldwide, ranging from 0.59% to 12% [2, 3]. A meta-analysis has demonstrated that the peak age of IS in China is 14 to 15 years old, and the prevalence of IS in females is higher than in males [4]. Children with IS show a generalized low bone mineral density (BMD), which brings complications such as pain, fracture tendency, skeletal deformity, and abnormal bone development [5–7]. These problems greatly threaten the appearance and school activities of children with IS, but low BMD may be ignored in clinical practice because people focus on correcting deformity [8].

The risk of curve progression in IS children with low BMD was twice as high as those with normal BMD, suggesting that BMD may be one of the important prognostic factors for IS [9, 10]. Low BMD results in changes in bone mechanical properties, such as increased fragility and microfractures of vertebrae, and may lead to spinal asymmetry then [11]. IS children with low BMD also showed impaired ossification and bone growth, which may be associated with inconsistent degrees of curvature at different spinal sites, affecting the spinal structure and leading to the progression of IS [12].

There are many clinical interventions for children with IS to correct spinal deformity, including conservative and surgical protocols [13, 14]. However, therapeutic exercise, bracing, or surgical treatment effectiveness is strongly associated with BMD [15, 16]. For example, the mechanism of the brace is based on the external force that prevents the spine from collapsing, but the correction effect may be altered due to low BMD in children with IS [15, 17]. Some studies also suggested that surgical intervention was not recommended for IS children with severe osteoporosis, as low BMD is related to unstable spinal fusion [18]. As a result, ignorance of low BMD may influence the effectiveness of spinal curve correction and may even lead to intervention failure [19].

Osteoporosis is diagnosed when BMD is 2 or 2.5 standard deviations (SD) below the age and gender-matched standardized mean value. The prevalence of osteoporosis is reported to be higher in children with IS than in healthy controls [20]. Low BMD or osteoporosis in children with IS may be related to multiple factors, including abnormalities of bone metabolism regulated by congenital genetic defects [21] or endocrine factors [22]. As an issue closely related to living habits, low BMD may be mediated by lifestyle-related factors [23]. Low BMD in children with IS may be related to a variety of factors, while there is no sound theory. Although genetics, endocrines, and lifestyle-related factors are currently hotspots in the research area of bone metabolism in children with IS, no study summarizes the potential risk factors related to low BMD in children with IS. The summary of risk factors related to low BMD may contribute to preventing osteoporosis in children with IS and provide implications for future research regarding the intervention on osteoporosis in children with IS. Therefore, this scoping review aims to summarize the existing literature on the genetics, endocrines, and lifestyle factors related to low BMD in children with IS.

## Methods

This scoping review was conducted under the guidance of Preferred Reporting Items for Systematic reviews and Meta-Analyses extension for Scoping Reviews (PRISMA-ScR) [24] and followed steps including study retrieval, evidence screening, and evaluation, data extraction, and synthesis of findings.

### Stage 1 Study retrieval

Studies were limited to English and Chinese language and published before January 2022. One reviewer searched PubMed, Web of Science Core Collection, Embase, Cochrane Library, and Cumulative Index to Nursing and Allied Health Literature (CINAHL). Completed initial search for articles by January 20^th^, 2022. We retrieved risk factors for osteoporosis in children with IS using keywords or subject headings, including “idiopathic scoliosis”, “IS”, “bone mineral density”, “BMD”, “risk factor”, and their synonyms.

### Stage 2 Evidence screening and evaluating

A comprehensive screening was conducted for the retrieved articles. Three reviewers (Y.Y., Z.C., Z.H.) screened the titles and abstracts of the retrieved articles independently to determine their relevance to the topic and assess their eligibility. They would check the full text of an article if necessary. Any disagreement would be solved by a discussion with a fourth reviewer (X.Z.). Eligibility criteria are shown in Table 1.

**Table 1:** The eligibility criteria

The inclusion criteria	
Patients	Children with IS and low BMD
Exposure	Studies on BMD, bone metabolism, or bone morphology
Comparison	Children with IS and normal BMD, or healthy people
Outcomes	(1) BMD measured by DXA, HR-pQCT, quantitative ultrasound or other quantitative instruments. And (2) Genetic/endocrine/lifestyle-related factors linked to bone metabolism
Research types	Observational studies or randomized controlled studies
The exclusion criteria	
Patients	(1) Children without IS; (2) Average age above 18
Comparison	Average age above 18
Outcomes	Studies that do not measure BMD and bone metabolism
Research types	Case studies, or animal studies

*IS* idiopathic scoliosis, *BMD* bone mineral density, *DXA* dual-energy x-ray absorptiometry, *HR-pQCT* high-resolution peripheral quantitative computer tomography

### Stage 3 Quality assessment

Methodological quality was assessed in terms of different study types. To evaluate the risk of bias in randomized controlled trials (RCTs) and clinical controlled trials, we utilized version 2 of the Cochrane risk-of-bias tool for randomized trials (RoB 2) [25]. Rating case–control studies using the Newcastle–Ottawa Scale (NOS) [26] and the assessment of multiple systematic reviews (AMSTAR 2) for systematic reviews [27]. The NOS uses a semi-quantitative assessment of the star system to measure the quality of the study. It is divided into three sections: Selection, Comparability, and Exposure, with a total score of nine. Y.Y. and Z.C. evaluated the risk of bias in the retrieved articles independently, and disputes were discussed and weighed by Q.D.

### Stage 4 Data extraction and synthesis

The data extraction was conducted by three reviewers (Y.Y., Z.C., Z.H.) and the results of the preliminary extracted data would be submitted to a fourth reviewer (Q.D.) for re-examining. Any differences would be further discussed to ensure consistency among reviewers. We extracted data from the enrolled articles, including author, publication year, number of publications, sample size, and risk factors related to low BMD (such as genetic, endocrine, or lifestyle-related factors). A qualitative approach was employed to describe and synthesize the risk factors because of the methodological heterogeneity of the included studies.

## Results

A total of 1498 records, including four from the reference lists, were first retrieved from the five databases. After removing 419 duplicated and 1019 unrelated studies, we finally included 56. There were 30 studies [6, 28–56] on genetic risk factors related to low BMD in children with IS, eight studies on endocrine risk factors [57–64], and 18 studies [5, 65–81] on lifestyle-related risk factors. A total of 10,768 participants with IS were included in the 56 included studies, consisting of 295 male patients with IS in 14 studies [30, 31, 33, 38, 40, 43, 44, 48, 51, 56, 63–66].

Tables S1, S2, and S3 show our assessment of the risk of bias. The overall bias of RCTs and clinical controlled trials was between some concerns and high. According to the methodological quality evaluation results of AMSTAR 2, three systematic reviews have critically low overall confidence. The risk of bias score of 50 case–control studies ranged from five to nine points, with 12 studies scoring nine points, indicating the quality of included studies was generally good.

### Part I: Bone mineral density

We found five studies [82–86] investigating the prevalence of osteoporosis in children with idiopathic scoliosis (IS). The results showed that the prevalence of osteoporosis in children with IS was 4.1% to 28.1%, with a median prevalence of 12.2%. In children without IS, the prevalence of osteoporosis ranged from 0% to 3.7% with a median prevalence of 2.1%.

### Part II: Genetic factors

Table 2 shows the possible genetic mechanisms underlying low BMD in children with IS. BsmI is an important factor in BMD, which is made up of two nucleotide variants A-G in the genotype of the vitamin D receptor (VDR) gene's eight introns [50]. Our results showed that low BMD might be correlated to VDR BsmI rs1544410 polymorphism in Asian children with IS [6, 28, 31, 50]. Osteoprotegerin (OPG) is a receptor activator of nuclear factor kappa-B ligand (RANKL) decoy receptor that prevents RANKL from binding to its receptor receptor activator of nuclear factor kappa-B (RANK), preventing osteoclast development and activation [40]. Ten included studies [30, 32, 36, 40, 42, 44, 48, 51, 52, 54] showed that G → C mutation in OPG gene site 1181 and T single nucleotide polymorphism in adiponectin gene site rs7639352 might be associated with low BMD. In addition, BMD in children with IS may be decreased by the knockdown or overexpression of miR-145/β-catenin via inhibiting osteocyte function. Low BMD in AIS may be related to the dropped osteogenic differentiation ability in the mesenchymal stem cells (MSCs) mechanism [39, 55, 56]. Three studies showed that low Runx2 expression might be related to low BMD [43–45]. G-c polymorphism at the -174 and -572 sites of the Interleukin-6 (IL-6) gene promoter was associated with low BMD, and adiponectin may be a mediator in this pathway of bone development [33, 34, 51].

**Table 2:** Influence of genetic factors on bone health in idiopathic scoliosis

Gene/signal pathway	Number of studies	The sample size of IS patients	Outcome measures relate to bone	Results	Research type
VDR gene [6, 28, 31, 50]	4	717	BMD (*n *= 4)	VDR BsmI rs1544410 polymorphism was susceptive to low BMD in Asians	Observational study (*n* = 2) Systematic review (*n* = 2)
RANK/RANKL signal pathway [30, 32, 36, 40, 42, 44, 48, 51, 52, 54]	10	525	Bone metabolism parameters (*n* = 3) BMD (*n* = 5) Bone development endocrine (*n* = 1) Bone morphology parameters (*n* = 1)	T single nucleotide polymorphism in Adiponectin gene site rs7639352 and G → C mutation in OPG gene site 1181 may be associated with low BMD	Observational study (*n* = 10)
MSCs [39, 55, 56]	3	35	BMD (*n* = 3)	The mechanisms underlying low BMD in AIS may be related to the dropped osteogenic differentiation ability	Observational study (*n* = 3)
Runx2 [43–45]	3	78	BMD (*n* = 2)	Low Runx2 expression may related to low BMD	Observational study *(n* = 3)
IL-6 related genes [33, 34, 51]	3	472	Bone metabolism parameters (*n* = 1) BMD (*n* = 2) Bone morphology parameters (*n* = 1)	G-c polymorphism at the -174 and -572 sites of the IL-6 gene promoter was associated with low BMD Adiponectin may be a key factor in this pathway	Observational study (*n* = 3)
miR-145/β-catenin [35, 53]	2	22	Bone metabolism parameters (*n* = 2)	Knockdown or overexpression of miR-145/β-catenin inhibits osteocyte function	Observational study (*n* = 2)

*VDR* vitamin D receptor, *BMD* bone mineral density, *RANK* receptor activator of nuclear factor *kappa-B; RANKL* receptor activator of nuclear factor kappa-B ligand, *OPG* Osteoprotegerin, *MSCs* mesenchymal stem cells, *IL-6* Interleukin-6

The genes related to low BMD in children with IS may consist of WNT16 [29], LBX1 in muscle and bone [37], or the insulin-like growth factor-I gene [49]. Our results also showed a potential relationship between low BMD and genetic variants of CHD7 [47], estrogen receptor gene polymorphism [46], or single nucleotide polymorphism [38].

### Part III: Endocrine factors

Table 3 shows the influence of endocrine factors on BMD in children with IS. Many endocrine factors mediate bone development and our results showed that the endocrine factors associated with low BMD in children with IS consisted of serum leptin and soluble leptin receptor (SLR) [57, 59–62], sclerostin [58], ghrelin [62], and IL-6 [63]. Nontargeted plasma metabolite analysis also showed that there might be an association between oxoglutarate, L-arginine arginine, N-acetylaspartate, citrate, and bone mass in children with IS [64].

**Table 3:** Influence of endocrine factors on bone health in idiopathic scoliosis

Endocrine factors	Number of studies	Sample size	Other outcome measures	Results	Research type
Serum leptin [59]	1	120	Anthropometric data	Decrease in serum leptin Correlations between leptin and the BMI, and BMC/BMD	Observational study
SLR [57, 60–62]	4	486	HR-pQCT parameters(*n *= 3) Anthropometric parameters (*n* = 1)	Decrease in serum leptin Increase of SLR Correlations between leptin and bone-related measures	Observational study (*n* = 3) Systematic review (*n* = 1)
Serum sclerostin [58]	1	47	Anthropometric parameters DXA/HR-pQCT parameters	Increase the level of serum sclerostin Correlation between serum sclerostin and cortical volumetric BMD and thickness	Observational study
Serum ghrelin [62]	1	132	/	Increase of ghrelin	Systematic review
IL-6 [63]	1	36	Anthropometric parameters BMD	Correlation between IL-6 and BMD	Observational study
Nontargeted plasma metabolites [64]	1	16	Anthropometric parameters BMD	Correlation between oxoglutarate, L-arginine, arginine, N-acetylaspartate, citrate, and BMI/BMD	Observational study

*BMI* body mass index, *BMC* bone mineral content, *BMD* bone mineral density, *SLR* soluble leptin receptor, *HR-pQCT* high-resolution peripheral quantitative computed tomography, *DXA* dual-energy X-ray absorptiometry, *IL-6* Interleukin-6

### Part IV: Lifestyle-related factors

Lifestyle-related factors related to BMD include serum vitamin D/25-hydroxyvitamin D (vitamin D/25(OH)D) [65–68, 72, 73], calcium intake [5, 69, 72, 73, 75–79, 81], physical activity [69, 72–74, 76, 77, 79, 80], grip strength [70], whole-body vibration [71]. Although no significant differences in diary calcium intake between the IS group and the control group [5, 69, 72, 73, 75–79, 81], calcium intake and serum vitamin D/25(OH)D levels may be correlated with low BMD [5, 65–69, 72, 73, 75–79, 81]. Improving grip strength may bring extra benefits to BMD in children with IS [70]. But the effect of whole-body vibration on BMD in children with IS needs further exploration [71]. The influence of lifestyle-related factors on bone health in children with IS is shown in Table 4.

**Table 4:** Influence of lifestyle-related factors on bone health in idiopathic scoliosis

Lifestyle factor	Number of studies	The sample size of IS patients	Outcome measures relate to bone	Conclusions	Research type
Serum vitamin D/25(OH)D [65–68, 72, 73]	5	752	BMD (*n* = 5) Alkaline phosphatase (*n* = 2) Serum calcium (*n* = 2), Phosphorus (*n* = 1)	Decrease of vitamin D/25 (OH) D Correlation between vitamin D/25(OH)D and serum calcium Correlation between vitamin D/25 (OH) D and BMD	Observational study (*n* = 5)
Calcium intake [5, 69, 72, 73, 75–79, 81]	10	4362	DXA/HR-pQCT parameters (*n* = 10) Alkaline phosphatase (*n* = 2) Urinary deoxypyridinoline (*n* = 2)	Correlation between calcium intake and BMD Increase of calcium intake may improve BMD	Observational study (*n* = 9) Randomized controlled study (*n* = 1)
Physical activity [69, 72–74, 76, 77, 79, 80]	8	2542	BMD (*n* = 7)	Correlation between physical activity and BMD	Observational study (*n* = 7) Randomized controlled study (*n* = 1)
Grip strength [70]	1	142	HR-pQCT parameters (*n* = 1)	Correlation between grip strength and cortical area, cortical thickness, bone mechanical properties, and structure model index	Observational study
Whole body vibration [71]	1	30	HR-pQCT parameters (*n* = 1)	/	Clinical controlled trial

*BMD* bone mineral density, *vitamin D/25(OH) D* vitamin D/25-hydroxyvitamin D, *DXA* dual-energy X-ray absorptiometry, *HR-pQCT* high-resolution peripheral quantitative computed tomography

## Discussion

In this study, we investigated the possible risk factors, including genetic, endocrine, and lifestyle-related factors of low BMD in children with IS. A total of 6 genetic factors are mentioned, including the VDR gene, RANK/RANKL signal pathway, the function of MSCs, Runx2, IL-6 related gene, and miR-145/β-catenin pathway. Endocrine factors included serum leptin and SLR, sclerostin, ghrelin, IL-6, and some nontargeted plasma metabolites, which may be associated with low BMD in children with IS. In addition, lifestyle-related factors such as vitamin D level, calcium intake, physical activity, and body composition also affect bone health in children with IS.

### Bone mineral density

There might be a trend that the BMD of children with IS is significantly lower than that of healthy individuals [85, 87]. In a general way, BMD lagging the same age and gender population by 1 to 2 SD was defined as osteopenia, while a lag beyond 2 or 2.5 SD was defined as osteoporosis [88]. Notably, the prevalence of osteoporosis in children with IS was much higher than in healthy controls, suggesting that osteoporosis may be a comorbidity in IS patients involved in the onset or progress of IS. From this perspective, this review extensively retrieves multiple factors that provide evidence for osteoporosis in children with IS. However, one study showed no significant differences in femoral neck BMD Z-score between children with IS and the control group [89]. The possible reason for the difference is that in this study, the control group was children who needed orthopedic surgery and might also have low BMD.

### Genetic factors

VDR is a ligand-dependent nuclear transcription factor [90]. The VDR gene is located on chromosome 12q13 and can rapidly mediate the function of vitamin D [31]. It has been reported that VDR gene polymorphism was related to serum vitamin D concentration, and BsmI site polymorphism had an important impact on VDR activity [91]. Existing studies suggest that the frequency of the Bb phenotype at the BsmI site of the VDR gene is significantly increased in children with IS, but whether this is the cause of low BMD is still controversial.

RANKL activates osteoclasts by binding to RANK molecules on the cell membrane of osteoclasts, while OPG competes with RANK to bind RANKL to inhibit the function of osteoclasts and their precursors [92, 93]. The balance of RANKL/RANK and OPG is vital in chronic arthritis, hormone-induced osteoporosis, etc. The serum RANKL concentration in children with scoliosis is abnormally increased, while the OPG level is similar to that of the average population. When the ratio of RANKL/RANK to OPG is unbalanced, osteoclasts are excessively activated, and bone resorption is enhanced, ultimately leading to a decrease in BMD. It was also found that the expression of Mir-145 and adiponectin was up-regulated in IS, the former inhibiting the production of OPG while the latter stimulating the production of RANKL [94].

MSCs play a key role in bone growth, they can differentiate into osteoprogenitor cells and cartilage progenitor cells and can affect osteoclast function by regulating RANKL and OPG [95]. At present, it is believed that the osteogenic differentiation ability of MSCs in children with IS is decreased may be due to various reasons. For example, the expression of pyruvate kinase isoenzyme M2 (PKM2) in stem cells is up-regulated, which inhibits cell differentiation [55]. ERK1/2 signaling is activated by the downregulation of heat shock protein HSP70, which promotes osteogenic differentiation and proliferation of MSCs [56]. Additionally, Runx2 is a key transcription regulator of osteoblast differentiation and maturation, and its downregulation also inhibits the osteogenic differentiation of MSCs [96].

In bone tissue, IL-6 is produced by osteoblasts, monocytes, and T cells and plays a prominent role in bone metabolism by promoting osteoclastogenesis and osteoclast activity. In the IL-6 gene promoter region, the -174G/C polymorphism and the -572G/C polymorphism alters levels of plasma IL-6. Because the promoter activity with the C allele is 40% lower than that with the G allele, osteoporosis is common in the GG phenotypic population [97]. The -572G allele was shown to be much more common in the Asian IS population, while the -174 locus polymorphism was found to be more prevalent in Europeans [97].

### Endocrine factors

Leptin is involved in bone remodeling through direct or indirect means, acting on the development of bone diseases such as osteoporosis [98]. Studies proposed that low serum leptin and low SLR levels might related to low BMD [59, 62]. Leptin is produced by white adipocytes, and binds to leptin receptors on the membranes of osteoblasts and chondrocytes, stimulating signaling pathways such as JAK2, STAT3, and MAPK to affect bone cell development and differentiation [99]. Leptin regulates the hypothalamic-pituitary function and affects the levels of thyroid hormone, growth hormone, cortisol, and steroids, and further regulates bone metabolism [99]. The possible mechanism of leptin in regulating BMD may be through the OPG/RANKL signaling pathway that enhances osteoblasts and inhibits osteoclastogenesis [100]. In contrast, the SLR binds to circulating leptin and suppresses signaling pathways [101]. At present, it is believed that the mechanism of leptin impacting BMD in children with IS may be multi-faceted, including insufficient leptin secretion, abnormal expression of SLR, and low sensitivity of bone tissue to leptin signaling [102].

IL-6 affects the differentiation and function of osteoclasts, and studies have shown that IL-6 is significantly associated with hip BMD in women with insufficient calcium intake [103]. IL-6 can be produced directly in cells by lipopolysaccharide, which stimulates IL-6 by inducing a series of intermediate cytokines IL-1 and tumor necrosis factor (TNF) [104]. Excessive secretion of IL-6 may be one of the essential factors causing low BMD in children with IS [63].

### Lifestyle-related factors

Identifying the underlying risk factor of osteoporosis in children or teenagers and eliminating risk factors that lead to low BMD should be the initial steps in treatment. Bisphosphonates have been demonstrated to have therapeutic effects on osteoporosis in children by inhibiting the mevalonate pathway, but their safety is unknown. Potential side effects include inhibiting cartilage production [105], raising the chance of atypical femoral bone [106], and interfering with tooth eruption [107].

Nutrition is the most effective means of optimizing peak BMD [108]. The putative mechanism by which calcium intake affects bone health is through increased BMD, which also increases muscle and increases activity [109]. While the median calcium intake of children with IS was 517.7 mg/d, which was only 50% of the reference intake [69]. Vitamin D and calcium supplements are interventions for low BMD patients [110].

Exercise reduces the production of inflammatory factors such as IL-6 and TNF-a and increases the secretion of cytokines that inhibit bone resorption, such as IL-2 and IL-10 [111]. Studies showed that girls with IS may be more reluctant to exercise than other children [69]. Long-term weight-bearing physical activity programs have been proven to improve BMD and structural characteristics without causing harm, and the benefits last over time [112]. A healthy diet and regular exercise are preventive factors against osteoporosis in children with IS. Children with IS, on the other hand, have low body fat and lean body weight. Although the link between this inclination and low BMD has yet to be established, we believe that a balanced protein and fat consumption will benefit the skeletal development of children with IS [113].

### Factors connection

The association between genetics, endocrines, and lifestyle factors may help to understand the pathogenesis of osteoporosis in children with IS.

Approximately 60–80% of the variation in bone structure was caused by genetic factors and developed before adolescence [114]. Genetic factors may regulate BMD by influencing hormone secretion and hormone receptors related to bone synthesis and absorption [33]. For example, the variation of IL-6 related genes may affect the BMD via the endocrine pathway in children with IS because IL-6 is a critical cytokine that promotes bone resorption. A study showed that G-c polymorphism at the -174 sites of the IL-6 gene promoter in children with IS might be associated with serum IL-6 levels [33]. When IL-6 is excessively secreted, it will disrupt the balance of bone metabolism and cause low BMD in children with IS [63]. In addition, VDR BsmI polymorphism plays a key role in bone metabolism regulation [50]. The VDR gene codifies instructions for making VDR protein respond to 25(OH)D [115]. After combining with 25(OH)D, VDR regulates the proliferation and differentiation of osteoblasts, osteoclasts, and chondrocytes [90]. Mutations in the VDR gene may lead to abnormal expression or non-expression of VDR [28]. For those children with IS who have deficits in the VDR gene, low BMD may be detected even if serum 25(OH)D is in the normal range [6].

Some lifestyles will disrupt the endocrine balance, leading to bone synthesis or absorption disorders. Leptin levels are positively correlated with BMD in children with IS [59]. Studies have shown that physical activity, especially moderate-vigorous physical activity, may promotes the body's responsiveness to leptin [116, 117]. Long-term pro-inflammatory dietary patterns like trans-hydrogenated fats and fried food can lead to inflammation [118]. One study showed that people who have taken pro-inflammatory food for a long time had a lower level of the lumbar spine and total hip BMD than those who have a healthy diet [119]. One of the possible explanations may be that long-term pro-inflammatory dietary patterns may stimulate excessive secretion of IL-6, which reduces the BMD of children with IS.

### Clinical implications

A comprehensive screening protocol should be designed based on the risk factors related to low BMD found in this review because a wide-ranged screening may be an effective step to improve bone strength and prognosis. The screening protocol may include risk gene analysis (e.g., VDR gene and IL-6 gene), endocrine test (e.g., serum leptin and SLR, sclerostin, ghrelin, and IL-6), and review of daily living (e.g., nutrition and physical activities). Lifestyle-related factors might be appropriate interventional targets for low BMD in children with IS, as there are many rehabilitation programs for lifestyle modification. Future studies may be needed to determine which type and frequency of physical activity are best for improving BMD in children with IS. Calcium and vitamin D are necessary for treating low BMD, and daily calcium and vitamin D intakes for children with IS needs to be investigated.

Osteoporosis refers to people with low BMD, altered bone microarchitecture, increased fragility, and fracture risk [120]. This definition is consistent with the effects of the three factors summarized in this review. However, according to the latest criteria on diagnosing osteoporosis in children and adolescents, a history of clinically meaningful fractures is mandatory, in addition to a low BMD score of less than -2.0. Otherwise, the children should present a nontraumatic vertebral compression fracture [121]. Our included articles have insufficient information to draw relationships between children with IS and osteoporosis. Although it is clear that these risk factors are associated with low BMD and changes in bone quality in children with IS. More research on this population and osteoporosis is needed in the future.

## Conclusions

This scoping review showed that BMD was generally lower in children with IS than in asymptomatic controls. Genetic, endocrine, and lifestyle-related factors might be associated with low BMD in children with IS. Bone synthesis or absorption may be directly regulated by endocrine factors, while genetic and lifestyle-related factors may influence BMD via the endocrine pathway. Comprehensive screening for low BMD risk factors may be reasonable to prevent osteoporosis and progression in children with IS. Future studies may consider targeted intervention protocols, including lifestyle modifications, to improve the low BMD in children with IS.

## Supplementary Information

**Additional file 1. Table S1. **Risk of bias Assessmentby Newcastle-Ottawa Scale for Case-Control Studies. **Table S2.** Risk of BiasAssessment by RoB 2 for Randomized Controlled Trials. **Table S3.** Risk of BiasAssessment by AMSTAR 2 for Systematic Reviews.

## Data Availability

The datasets used and/or analyzed during the current scoping review are available from the corresponding authors on reasonable request.

## Abbreviations

IS: Idiopathic scoliosis
BMD: Bone mineral density
IL-6: Interleukin-6
SD: Standard deviation
PRISMA-ScR: Preferred Reporting Items for Systematic reviews and Meta-Analyses extension for Scoping Reviews
CINAHL: Cumulative Index to Nursing and Allied Health Literature
RCTs: Randomized controlled trials
RoB 2: Version 2 of the Cochrane risk-of-bias tool for randomized trials
NOS: Newcastle–Ottawa Scale
AMSTAR 2: Assessment of multiple systematic reviews
VDR: Vitamin D receptor
OPG: Osteoprotegerin
RANKL: Receptor activator of nuclear factor kappa-B ligand
RANK: Receptor activator of nuclear factor kappa-B
MSCs: mesenchymal stem cells
SLR: soluble leptin receptor
vitamin D/25(OH)D: vitamin D/25-hydroxyvitamin D
PKM2: pyruvate kinase isoenzyme M2
TNF: tumor necrosis factor
