# Supplementary material for: Risk factors associated with low bone mineral density in children with idiopathic scoliosis: a scoping review
Source: BMC Musculoskelet Disord. 2023 Jan 20;24:48. doi: 10.1186/s12891-023-06157-8 (PMC9854192; doi:10.1186/s12891-023-06157-8)
Supplement: Supplementary file 1 — Additional file 1. Table S1. Risk of bias Assessmentby Newcastle-Ottawa Scale for Case-Control Studies. Table S2. Risk of BiasAssessment by RoB 2 for Randomized Controlled Trials. Table S3. Risk of BiasAssessment by AMSTAR 2 for Systematic Reviews. [file 12891_2023_6157_MOESM1_ESM.docx]

**Table S1. Risk of bias Assessment by Newcastle-Ottawa Scale for Case-Control Studies.**

|  | **Article, Year** | **Selection** | | | | **Comparability** | **Exposure** | | |
| --- | --- | --- | --- | --- | --- | --- | --- | --- | --- |
|  |  | **1) Is the case definition adequate?** | **2) Representativeness of the cases** | **3) Selection of Controls** | **4) Definition of Controls** | **1) Comparability of cases and controls** | **1) Ascertainment of exposure** | **2) Same method of ascertainment for cases and controls** | **3) Non-Response rate** |
| 1 | Alsiddiky A, 2020 (65) | ✮ | ✮ | - | - | ✮ | ✮ | ✮ | - |
| 2 | Balioglu M, 2017 (66) | ✮ | ✮ | - | ✮ | ✮✮ | ✮ | ✮ | - |
| 3 | Batista R, 2014 (67) | ✮ | ✮ | - | ✮ | - | ✮ | ✮ | - |
| 4 | Catan L, 2020 (68) | ✮ | ✮ | ✮ | ✮ | ✮✮ | ✮ | ✮ | ✮ |
| 5 | Chen W, 2008 (28) | ✮ | ✮ | ✮ | ✮ | - | ✮ | ✮ | - |
| 6 | Cheng J, 2007 (69) | ✮ | ✮ | ✮ | ✮ | ✮ | ✮ | ✮ | ✮ |
| 7 | Cheng K, 2021 (29) | ✮ | ✮ | ✮ | ✮ | ✮✮ | ✮ | ✮ | ✮ |
| 8 | Cheuk K, 2018 (70) | ✮ | - | - | ✮ | ✮✮ | ✮ | ✮ | - |
| 9 | Cheung C, 2006 (5) | ✮ | ✮ | ✮ | ✮ | ✮✮ | ✮ | ✮ | ✮ |
| 10 | Chiru M, 2011 (30) | ✮ | ✮ | - | ✮ | ✮✮ | ✮ | ✮ | - |
| 11 | Eun I, 2009 (32) | ✮ | ✮ | ✮ | - | ✮✮ | ✮ | ✮ | ✮ |
| 12 | Gao J, 2018 (33) | ✮ | ✮ | ✮ | ✮ | ✮✮ | ✮ | ✮ | ✮ |
| 13 | Lam E, 2015 (57) | ✮ | ✮ | - | ✮ | ✮✮ | ✮ | ✮ | ✮ |
| 14 | Lam T, 2013 (72) | ✮ | ✮ | - | ✮ | ✮ | ✮ | ✮ | ✮ |
| 15 | Lam T, 2015 (73) | ✮ | ✮ | - | ✮ | ✮✮ | ✮ | ✮ | ✮ |
| 16 | Lee J, 2010 (34) | ✮ | ✮ | ✮ | ✮ | ✮✮ | ✮ | ✮ | ✮ |
| 17 | Lee W, 2003 (75) | ✮ | ✮ | ✮ | ✮ | ✮✮ | ✮ | ✮ | ✮ |
| 18 | Lee W, 2005 (76) | ✮ | ✮ | ✮ | ✮ | ✮✮ | ✮ | ✮ | ✮ |
| 19 | Lee W, 2006 (77) | ✮ | ✮ | - | ✮ | ✮ | ✮ | ✮ | ✮ |
| 20 | Lee W, 2006 (78) | ✮ | ✮ | - | ✮ | ✮ | ✮ | ✮ | ✮ |
| 21 | Lee W, 2017 (58) | ✮ | ✮ | - | ✮ | ✮ | ✮ | ✮ | ✮ |
| 22 | Lee W, 2017 (35) | ✮ | ✮ | - | ✮ | ✮ | ✮ | ✮ | ✮ |
| 23 | Liu Z, 2008 (36) | ✮ | ✮ | - | ✮ | ✮ | ✮ | ✮ | ✮ |
| 24 | Man G, 2017 (37) | ✮ | ✮ | - | ✮ | ✮ | ✮ | ✮ | ✮ |
| 25 | Moon E, 2013 (38) | ✮ | ✮ | ✮ | ✮ | ✮✮ | ✮ | ✮ | ✮ |
| 26 | Park W, 2009 (39) | ✮ | ✮ | - | ✮ | ✮✮ | ✮ | ✮ | ✮ |
| 27 | Popa O, 2010 (40) | ✮ | ✮ | - | ✮ | - | ✮ | ✮ | - |
| 28 | Qiu X, 2007 (41) | ✮ | ✮ | ✮ | ✮ | ✮✮ | ✮ | ✮ | ✮ |
| 29 | Qiu Y, 2007 (59) | ✮ | ✮ | ✮ | ✮ | ✮✮ | ✮ | ✮ | ✮ |
| 30 | Suh K, 2010 (6) | ✮ | ✮ | - | ✮ | ✮✮ | ✮ | ✮ | ✮ |
| 31 | Suh K, 2007 (42) | ✮ | ✮ | ✮ | ✮ | ✮✮ | ✮ | ✮ | ✮ |
| 32 | Sun C, 2009 (43) | ✮ | ✮ | - | ✮ | ✮ | ✮ | ✮ | ✮ |
| 33 | Sun C, 2010 (44) | ✮ | ✮ | - | - | ✮ | ✮ | ✮ | - |
| 34 | Tam E, 2014 (60) | ✮ | ✮ | ✮ | ✮ | ✮✮ | ✮ | ✮ | ✮ |
| 35 | Tam E, 2013 (61) | ✮ | ✮ | - | ✮ | ✮✮ | ✮ | ✮ | ✮ |
| 36 | Tobias J, 2019 (80) | ✮ | ✮ | ✮ | ✮ | ✮✮ | - | ✮ | ✮ |
| 37 | Wang W, 2014 (45) | ✮ | ✮ | ✮ | - | ✮✮ | ✮ | ✮ | ✮ |
| 38 | Wu J, 2007 (46) | ✮ | - | ✮ | - | ✮✮ | ✮ | ✮ | ✮ |
| 39 | Wu J, 2005 (63) | ✮ | ✮ | - | ✮ | ✮✮ | ✮ | ✮ | ✮ |
| 40 | Wu Z, 2021 (47) | ✮ | ✮ | - | ✮ | ✮✮ | ✮ | ✮ | ✮ |
| 41 | Xiao L, 2021 (64) | ✮ | ✮ | - | ✮ | ✮✮ | ✮ | ✮ | ✮ |
| 42 | Xiao L, 2020 (48) | ✮ | ✮ | - | ✮ | ✮✮ | ✮ | ✮ | ✮ |
| 43 | Yang G, 2020 (81) | ✮ | ✮ | ✮ | - | ✮✮ | ✮ | ✮ | - |
| 44 | Yeung H, 2006 (49) | ✮ | ✮ | ✮ | ✮ | ✮ | ✮ | ✮ | - |
| 45 | Zhang H, 2019 (51) | ✮ | ✮ | - | ✮ | ✮✮ | ✮ | ✮ | ✮ |
| 46 | Zhang J, 2017 (52) | ✮ | ✮ | - | ✮ | - | ✮ | ✮ | ✮ |
| 47 | Zhang J, 2018 (53) | ✮ | ✮ | - | ✮ | ✮✮ | ✮ | ✮ | ✮ |
| 48 | Zhou S, 2012 (54) | ✮ | ✮ | - | ✮ | ✮✮ | ✮ | ✮ | ✮ |
| 49 | Zhuang Q, 2011 (55) | ✮ | ✮ | - | ✮ | ✮ | ✮ | ✮ | ✮ |
| 50 | Zhuang Q, 2016 (56) | ✮ | ✮ | - | ✮ | ✮ | ✮ | ✮ | ✮ |

**✮: Stars would be assigned if the items met the requirements of low risk of bias.**

**Table S2. Risk of Bias Assessment by RoB 2 for Randomized Controlled Trials.**

|  | **Article, Year** | **Randomization Process** | **Deviations From the Intended Interventions** | **Missing Outcome Data** | **Measurement Of the Outcome** | **Selection Of the Reported Result** | **Overall Bias** |
| --- | --- | --- | --- | --- | --- | --- | --- |
| 1 | Christen P, 2017 (71) | N/N/NI  High | Y/Y/N/NA/NA/PN/PN  Some concerns | NI/N/N  Low | N/PN/Y/N  Low | NI/Y/N  High | High |
| 2 | Lau RW, 2021 (74) | PY/N/N  High | Y/Y/NI/NA/NA/PY/NA  Some concerns | N/N/NI/NI  High | N/N/NI/N  Low | PY/Y/PN  High | High |
| 3 | Normand E, 2018 (79) | NI/PY/PN  Low | PY/PY/N/NA/NA/PY/NA  Low | N/N/PN  High | N/N/PY/PN  Low | PY/NI/NI  Some concerns | Some concerns |

Y: Yes; N: No; PY: Probably Yes; PN: Probably No; NI: No Information; NA: Not Applicable.

**Table S3. Risk of Bias Assessment by AMSTAR 2 for Systematic Reviews.**

|  | **Article, Year** | **Item 1** | **Item 2** ^a^ | **Item 3** | **Item 4** ^a^ | **Item 5** | **Item 6** | **Item 7** ^a^ | **Item 8** | **Item 9** ^a^ | **Item 10** | **Item 11** ^a^ | **Item 12** | **Item 13** ^a^ | **Item 14** | **Item 15** ^a^ | **Item 16** |
| --- | --- | --- | --- | --- | --- | --- | --- | --- | --- | --- | --- | --- | --- | --- | --- | --- | --- |
| 1 | Dai J, 2018 (31) | Y | N | N | Y | Y | Y | N | Y | PY | N | Y | N | N | Y | Y | N |
| 2 | Wang Q, 2020 (62) | Y | N | N | PY | Y | N | N | Y | PY | N | Y | Y | Y | Y | N | Y |
| 3 | Yin X, 2018 (50) | Y | N | N | Y | N | Y | N | Y | N | N | Y | N | N | Y | N | Y |

^a^: AMSTAR 2 Critical Domains; Y: Yes; PY: Partial yes; N: No.
